# Integrating AI, RNA Vaccines, and CAR‐T Cells for Personalized Treatment

**DOI:** 10.1155/jimr/2275385

**Published:** 2026-09-01

**Authors:** Diala Haykal, Brigitte Dréno

**Affiliations:** ^1^ Centre Médical Laser Palaiseau, Palaiseau, France; ^2^ Immunology and New Concepts in ImmunoTherapy, Nantes Université, CNRS, INCIT, INSERM, UMR 1302/EMR6001, Nantes, France, inserm.fr

## Abstract

Oncology is undergoing a profound transformation driven by the convergence of artificial intelligence (AI), RNA‐based vaccines, and chimeric antigen receptor T‐cell (CAR‐T) therapies. Individually, these technologies have advanced cancer diagnosis, treatment, and patient stratification. AI‐driven approaches enhance drug discovery, optimize clinical trial design, and enable personalized therapeutic decision‐making. RNA vaccines provide a flexible platform for encoding tumor‐specific neoantigens, while CAR‐T therapies enable targeted immune‐mediated tumor cell elimination. Early‐phase clinical trials, particularly those combining RNA vaccines with immune checkpoint inhibitors, have demonstrated promising improvements in recurrence‐free survival (RFS) and immunogenicity. However, evidence supporting direct combinations of RNA vaccines and CAR‐T therapies remains largely preclinical or limited to early‐phase investigation and should therefore be interpreted with caution. This review explores how AI facilitates neoantigen discovery, RNA vaccine optimization, and CAR‐T cell engineering, and examines the emerging interplay between these modalities. While their integration represents a compelling framework for personalized oncology, significant challenges remain, including clinical validation, scalability, regulatory oversight, and equitable access.

## 1. Introduction

Cancer treatment has entered a new era, driven by technological innovation and biomedical advancements. Among the most transformative breakthroughs are AI‐powered algorithms, RNA‐based vaccines, and chimeric antigen receptor T‐cell (CAR‐T) therapies, each offering distinct capabilities in cancer diagnosis, treatment, and management. Individually, these technologies have demonstrated substantial potential. AI enhances drug discovery and patient‐specific treatment optimization through predictive modeling and large‐scale data analysis. RNA‐based vaccines provide a flexible platform for encoding tumor‐specific antigens, enabling personalized immunotherapeutic strategies. Meanwhile, CAR‐T cell therapy utilizes genetically engineered immune cells to target and eliminate cancer cells with high specificity.

While each modality has shown significant promise independently, their convergence represents an emerging paradigm in oncology. The integration of AI‐driven predictive modeling, RNA‐based immune priming, and CAR‐T‐mediated cytotoxic targeting may enable more adaptive and multimodal treatment strategies capable of addressing tumor heterogeneity and therapeutic resistance. However, despite this conceptual potential, clinical translation remains in evolution and requires further validation.

In this review, the term “artificial intelligence” (AI) is used to describe a spectrum of computational approaches applied to oncology, ranging from conventional bioinformatic pipelines to machine‐learning (ML) and deep‐learning (DL) models. Traditional computational methods rely on predefined statistical rules and deterministic algorithms, whereas ML approaches enable data‐driven pattern recognition and predictive modeling. DL methods, as a subset of ML, further extend this capability through multilayered neural network architectures capable of extracting complex features from high‐dimensional datasets. For clarity, we use “AI‐based” to refer specifically to ML‐ or DL‐driven approaches, while other methods are described as computational.

This review aims to explore the synergistic potential of combining AI, RNA‐based vaccines, and CAR‐T cell therapies in advancing oncology. This article examines their individual contributions, current clinical applications, emerging integration strategies, and the key challenges that must be addressed to facilitate their incorporation into routine clinical practice.

## 2. Methodological Note

This narrative review was informed by a structured literature search conducted across PubMed, Scopus, and the Web of Science. The final search was performed in March 2025. The search strategy combined the following terms: (“artificial intelligence” OR “machine learning”) AND (“RNA vaccine” OR “mRNA vaccine” OR “CAR‐T”) AND “cancer” or “oncology,” with database‐specific adaptations where appropriate.

A total of 221 records were identified, of which 187 were screened based on the title and abstract. Following full‐text assessment, 64 studies were included based on their relevance to the mechanistic, clinical, and regulatory intersections of AI, RNA‐based vaccines, and CAR‐T therapies. Priority was given to peer‐reviewed clinical trials, high‐impact reviews, and mechanistic studies.

## 3. AI’s Role in Cancer Immunotherapy

AI has emerged as a critical tool in cancer immunotherapy, transforming how treatments are designed, optimized, and delivered. By integrating genomic, transcriptomic, and proteomic data, AI‐based models enable the identification of tumor‐specific neoantigens, thereby supporting the development of personalized immunotherapies [1–4]. ML approaches are also increasingly used to predict patient responses, guide clinical trial enrollment, and optimize treatment strategies.

In the context of RNA‐based vaccines, AI facilitates neoantigen prioritization by predicting peptide–major histocompatibility complex (MHC) binding affinity and immunogenicity. Similarly, in CAR‐T cell therapy, AI contributes to antigen target selection and receptor design by modeling antigen‐receptor interactions and minimizing off‐target effects. These approaches enhance both the precision and efficiency of therapeutic development [5–10].

**Figure 1:**
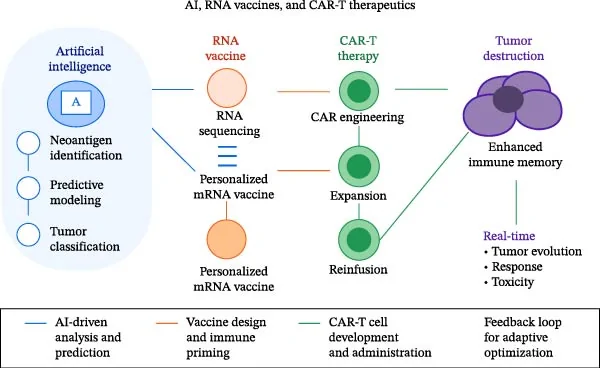
Synergistic Integration of AI, RNA vaccines, and CAR‐T therapeutics.

Illustrative examples of AI applications in oncology include platforms for protein structure prediction, digital pathology analysis, and drug discovery, which leverage ML and DL techniques to interpret complex biomedical data [1, 11]. Despite these advances, several challenges limit the clinical translation of AI in cancer immunotherapy. Model generalizability remains a major concern as many algorithms are trained on curated or homogeneous datasets that may not reflect real‐world population diversity or tumor heterogeneity. As a result, the model performance can decline when applied across different clinical environments. External validation using independent, multicenter datasets is therefore essential to ensure robustness and reproducibility. Furthermore, prospective clinical validation and integration into routine workflows remain limited, underscoring the need for standardized evaluation frameworks and regulatory oversight before widespread clinical adoption [12] (Figure 1).

## 4. RNA Technology in Cancer Vaccines

RNA‐based cancer vaccines have emerged as an emerging approach in oncology, leveraging the body’s own cells to produce tumor‐specific antigens that trigger an immune response [13–15]. Unlike traditional vaccines, which deliver weakened pathogens, RNA vaccines encode synthetic mRNA instructions, prompting cells to generate specific antigens that stimulate an immune response. These vaccines use synthetic mRNA sequences to instruct cells to generate proteins that mimic cancer antigens, prompting the immune system to recognize and destroy cancer cells [16, 17]. This adaptability allows for rapid customization based on a patient’s tumor profile, making personalized cancer vaccines feasible at scale. Companies like Moderna and BioNTech, pioneers in mRNA vaccine development, are applying this technology to cancer treatment, with several clinical trials underway targeting melanoma, lung cancer, and pancreatic cancer [18–20]. AI‐driven antigen selection models have further enhanced the precision of mRNA vaccine design, enabling faster development of highly personalized cancer vaccines [21–23].

A key example is the Phase 2b KEYNOTE‐942 trial evaluating the personalized mRNA‐4157 (V940) vaccine in combination with pembrolizumab in patients with resected high‐risk melanoma. This study demonstrated a significant improvement in recurrence‐free survival (RFS), with 18‐month RFS rates of 79% in the combination group compared to 62% with pembrolizumab alone, corresponding to a hazard ratio of 0.56 (95% CI: 0.31–0.99) [24]. These findings highlight the ability of personalized mRNA vaccines to enhance neoantigen‐specific T‐cell responses and improve disease control. However, evidence for overall survival benefit remains immature, and longer follow‐up is required to confirm the durability of effect.

## 5. CAR‐T Cell Therapy: A Revolutionary Addition

CAR‐T cell therapy represents a major advancement in cancer immunotherapy, particularly in hematologic malignancies. This approach involves the genetic engineering of patient‐derived T cells to express chimeric antigen receptors that recognize and eliminate tumor cells with high specificity. Despite its clinical success, several limitations remain, particularly when extending its application to solid tumors [25–27].

A critical challenge is the management of treatment‐related toxicity. Cytokine release syndrome (CRS) and immune effector cell‐associated neurotoxicity syndrome (ICANS) are among the most common and potentially severe adverse events associated with CAR‐T therapy, resulting from excessive immune activation and systemic cytokine release [9]. Early recognition and management strategies have significantly improved the safety profiles and feasibility of treatment. Gottschlich et al. demonstrated how AI‐driven single‐cell transcriptomic analysis advances CAR‐T cell development for acute myeloid leukemia. Their approach enabled rapid antigen target identification by analyzing over 500,000 single cells, uncovering targets like CSF1R and CD86 that traditional methods might miss. AI also streamlines CAR design by simulating antigen‐CAR interactions, reducing lab‐based experiments [28]. This integration accelerates clinical translation while improving treatment precision and patient safety.

In addition to toxicity, manufacturing constraints remain a major barrier to scalability. Autologous CAR‐T production is time‐intensive, costly, and logistically complex, limiting accessibility and delaying treatment initiation. These challenges are particularly relevant when considering combination strategies with other modalities, such as RNA vaccines, which require coordinated therapeutic timing.

As of early 2025, allogeneic (“off‐the‐shelf”) CAR‐T approaches are progressing through early‐phase clinical development, aiming to overcome these limitations by enabling scalable and readily available cell products. Recent reviews highlight advances in gene editing strategies to reduce graft‐versus‐host disease and improve safety, although efficacy remains under active investigation [29].

## 6. AI‐Mediated Immunologic Reinforcement Loop: A Hypothesis‐Generating Framework

The proposed AI‐mediated immunologic reinforcement loop should be regarded as a hypothesis‐generating framework rather than an evidence‐based clinical model. It conceptualizes how AI could integrate tumor immunogenomic data, RNA vaccine design, CAR‐T engineering, and longitudinal immune monitoring into an adaptive therapeutic strategy [30–33].

Several components of this framework are already feasible in clinical or translational settings. These include computational neoantigen prediction, RNA vaccine design, CAR‐T antigen target selection, and posttreatment immune monitoring using tools such as sequencing, flow cytometry, or circulating tumor DNA analysis.

However, other elements remain experimental. These include real‐time therapeutic recalibration, closed‐loop adjustment of RNA vaccine composition, dynamic modification of CAR‐T parameters after infusion, and fully AI‐directed treatment adaptation. Such approaches require prospective validation, standardized biomarkers, and regulatory oversight before they can be implemented in routine oncology practice [34, 35].

Therefore, while the AI–RNA–CAR‐T loop provides a useful conceptual model for future precision oncology, current evidence supports only selected components of this pathway. The full closed‐loop system remains investigational [36–39].

## 7. Clinical Trials and Notable Advances

### 7.1. RNA Vaccine Plus Immune Checkpoint Inhibitors

The most robust clinical evidence to date involves the combination of personalized RNA vaccines with immune checkpoint inhibitors. The Phase 2b KEYNOTE‐942 trial evaluating mRNA‐4157 (V940) in combination with pembrolizumab in patients with resected high‐risk melanoma demonstrated a significant improvement in RFS, with 18‐month RFS rates of 79% in the combination arm versus 62% with pembrolizumab alone (hazard ratio: 0.56; 95% CI: 0.31–0.99) [40]. These findings support the role of RNA vaccines in enhancing neoantigen‐specific immune responses and improving disease control.

Clinical evidence supporting the integration of AI, RNA‐based vaccines, and CAR‐T cell therapies can be more clearly understood when grouped according to therapeutic modality combinations [32, 40, 41].

### 7.2. RNA Vaccine Plus Immune Checkpoint Inhibitors

The most robust clinical evidence to date involves the combination of personalized RNA vaccines with immune checkpoint inhibitors. The Phase 2b KEYNOTE‐942 trial evaluating mRNA‐4157 (V940) in combination with pembrolizumab in patients with resected high‐risk melanoma demonstrated a significant improvement in RFS, with 18‐month RFS rates of 79% in the combination arm versus 62% with pembrolizumab alone (hazard ratio: 0.56; 95% CI: 0.31–0.99) [24].

Similarly, in pancreatic cancer, Rojas et al. demonstrated that personalized RNA neoantigen vaccines combined with immunotherapy induced strong polyclonal T‐cell responses and were associated with disease stabilization, supporting the applicability of this approach beyond highly immunogenic tumors [42].

These findings support the role of RNA vaccines in enhancing neoantigen‐specific immune responses and improving disease control (Table 1).

**Table 1:** Computational and AI‐based modeling in RNA vaccine development and CAR‐T cell engineering in translational oncology.

Selected clinical and computational studies of personalized cancer immunotherapies					
Study/Trial (year)	Cancer type	Intervention/Design	Key outcomes	Computational/AI Relevance	Ref
KEYNOTE‐942 (2024)	High‐risk melanoma	Personalized mRNA‐4157 (V940) + pembrolizumab vs pembrolizumab alone	18‐mo RFS 79% vs 62%; ARR 17%; HR 0.56 (95% CI 0.31–0.99)	Demonstrates synergy between neoantigen‐based vaccination and checkpoint inhibition; antigen selection supported by computational prediction algorithms	[24]
Ott et al., Nature 2017	Melanoma	Personalized RNA neoantigen vaccine	Induced broad CD4^+^/CD8^+^ responses; durable disease control	Computational neoantigen prediction and prioritization based on sequencing and HLA‐binding algorithms	[43]
Rojas et al., Nature 2023	Pancreatic cancer	Personalized RNA neoantigen vaccine + ICI	Strong polyclonal T‐cell expansion; disease stabilization	Computational epitope prioritization using immunogenomic pipelines	[42]
Fu et al., Signal Transduct Target Ther. 2024	Solid tumors (various)	Oncolytic virus + T cells or mRNA vaccine	Synergistic tumor regression; enhanced antigen spreading	In silico modeling of combination immunotherapy strategies and immune network interactions	[35]
Gottschlich et al., Nat Biotechnol. 2023	Acute myeloid leukemia	Single‐cell transcriptomic CAR‐T development	Identified >500 000 cells → new targets (CSF1R, CD86)	Machine learning‐driven single‐cell analysis enabling antigen discovery and CAR‐T target selection	[28]
Daei Sorkhabi et al., Front Immunol. 2023	Solid tumors (melanoma, ovarian, lung)	CAR‐T monotherapy (Phase I/II trials)	ORR 7%–20%; median duration 3–6 months	Highlights limitations of CAR‐T in solid tumors; rationale for combinatorial strategies	[41]
Chen et al., Mol Ther. 2024	Preclinical solid tumors	mRNA‐LNP + CAR‐T integration	Improved antigen specificity; decreased off‐target toxicity	Computational optimization of lipid nanoparticle delivery systems and therapeutic design	[33]
Zhu et al., Adv Mater. 2025	Solid tumors	Engineered CAR‐T with enhanced infiltration	Increased tumor penetration and safety profile	Deep learningbased receptor‐antigen interaction modeling and structural optimization	[37]
Paniagua et al., iScience 2025	Multitumor	AI + spatial transcriptomics	Identification of tumor microenvironment niches	AI‐based spatial transcriptomic analysis enabling precision targeting of immunotherapy strategies	[39]

### 7.3. CAR‐T Cell Therapy in Hematologic Malignancies

CAR‐T therapies have demonstrated substantial efficacy in hematologic cancers, including leukemia, lymphoma, and multiple myeloma, with high response rates and durable remissions reported in multiple clinical trials. Outcomes are typically measured in terms of objective response rate (ORR), complete remission rates, and progression‐free survival, reflecting the established role of CAR‐T therapy in these settings. However, translation to solid tumors remains limited due to the tumor microenvironment barriers and antigen heterogeneity.

### 7.4. RNA Vaccine and CAR‐T Combinations (Emerging Evidence)

Evidence supporting the combination of RNA vaccines and CAR‐T therapies remains limited and is largely derived from preclinical studies and early‐phase investigations. These studies suggest potential synergistic effects, including enhanced antigen spreading, improved T‐cell persistence, and increased immune activation. However, clinical endpoints such as survival or durable response have not yet been consistently demonstrated, and further validation in prospective trials is required.

In other solid tumors, including pancreatic and colorectal cancers, emerging studies combining RNA‐based immunotherapy with checkpoint inhibition have reported improvements in immune response and disease stabilization. However, these outcomes are primarily based on immunologic endpoints and early clinical signals rather than confirmed survival benefits and should therefore be interpreted with caution.

AI may shorten clinical trial timelines by improving patient selection and automating screening processes.

## 8. Applications in Dermatologic Oncology

This section is included to illustrate how the integration of AI, RNA‐based vaccines, and CAR‐T therapies can be translated into clinically relevant models, particularly in highly immunogenic tumors such as melanoma, which serve as a key platform for the development and validation of advanced immunotherapeutic strategies.

### 8.1. AI in Skin Cancer Diagnosis and Treatment

AI‐powered dermoscopy and histopathology tools have dramatically improved the early detection of melanoma and nonmelanoma skin cancers by enhancing diagnostic accuracy and reducing interobserver variability. DL models are now capable of distinguishing between benign and malignant lesions with a performance comparable to that of expert dermatologists. These systems also support clinical decision‐making by predicting tumor aggressiveness and potential responsiveness to immunotherapy [3, 44].

Despite these advances, dermatologic AI tools continue to face racial and phototype bias due to the underrepresentation of darker skin tones (Fitzpatrick IV–VI) in training datasets. Studies have demonstrated significantly lower diagnostic accuracy for melanoma in darker skin types, largely attributable to the limited image diversity in algorithm development. Esteva et al. and Haenssle et al. both reported dermatologist‐level accuracy using DL, yet the datasets used consisted predominantly of lighter phototypes, underscoring persistent disparity in AI model generalizability [3, 44]. Recent analyses emphasize the urgent need for inclusive image repositories and algorithmic fairness audits to ensure an equitable diagnostic performance across diverse populations [45, 46].

### 8.2. RNA Vaccines in Melanoma

Melanoma has been at the forefront of mRNA vaccine development. Personalized RNA vaccines targeting patient‐specific neoantigens have demonstrated promising early clinical results, particularly when combined with immune checkpoint inhibitors. These approaches have been associated with improved RFS and enhanced tumor‐specific immune responses. For example, the combination of mRNA‐4157 (V940) with pembrolizumab has been shown to significantly delay recurrence in patients with high‐risk melanoma [43, 47].

### 8.3. CAR‐T and Adoptive Cell Therapy in Skin Cancer

While CAR‐T therapies have been more successful in hematologic malignancies, their application in solid tumors, including melanoma and Merkel cell carcinoma, remains under active investigation. Emerging strategies include the use of tumor‐infiltrating lymphocytes (TILs) and CAR‐modified immune cells. Computational and ML‐based approaches contribute to the identification of tumor‐associated surface markers and the optimization of CAR constructs to target skin‐specific oncogenic pathways [48, 49].

Early‐phase melanoma CAR‐T trials have reported ORRs ranging between 7% and 20%, with median response durations of 3–6 months, reflecting limited persistence and infiltration of engineered T cells in solid tumors. These outcomes were summarized in recent meta‐analyses and reviews evaluating CAR‐T efficacy in cutaneous melanoma and Merkel cell carcinoma [48, 49]. Consequently, combinatorial strategies, such as integrating CAR‐T therapy with mRNA‐based immune priming or immune checkpoint inhibition, are being explored to enhance response durability and overcome these barriers [35, 37].

### 8.4. Extension to Other Solid Tumors

The AI–RNA–CAR‐T triad can be extrapolated to aggressive cancers such as triple‐negative breast cancer (TNBC) and glioblastoma multiforme (GBM), where tumor heterogeneity limits durable therapeutic responses. Computational and ML‐based epitope mapping has identified recurrent neoantigens in TNBC (e.g., MUC1 and NY‐ESO‐1), which are currently investigated in RNA vaccine constructs [38]. In GBM, AI‐based spatial transcriptomics delineates immunosuppressive niches, informing CAR‐T reprogramming strategies to resist PD‐L1‐mediated exhaustion [39]. These findings highlight the translational adaptability of the proposed integrative model beyond dermatologic malignancies.

### 8.5. Managing Dermatologic Toxicities of Immunotherapy

AI‐based tools are also being developed to monitor and predict dermatologic adverse events associated with immune checkpoint inhibitors and CAR‐T therapies. These approaches may enable earlier detection, personalized management strategies, and improved treatment adherence, ultimately enhancing patient quality of life [50, 51].

Collectively, these developments underscore the role of dermatologic oncology as a translational model for evaluating integrative immunotherapy strategies, bridging mechanistic innovation with clinically relevant applications in solid tumors.

## 9. Current Research Trends

Current research in the integration of AI, RNA vaccines, and CAR‐T cell therapies is increasingly focused on improving precision, scalability, and translational applicability. Advances in multiomics integration, including genomics, transcriptomics, and spatial transcriptomics, are enabling more refined tumor characterization and therapeutic targeting. However, several cross‐cutting challenges persist. Model drift, driven by tumor evolution and dynamic biological variability, can reduce the long‐term predictive performance of AI systems. In parallel, limited access to high‐quality, diverse datasets constrains model training and exacerbates biases, while variability in sequencing pipelines and analytical methods contributes to reduced reproducibility across studies. These issues are further compounded by restricted data sharing and the fragmentation of proprietary datasets. Addressing these interconnected challenges through standardized benchmarking, external validation, and collaborative data‐sharing frameworks will be essential to ensure the reliable clinical implementation of AI‐driven oncology strategies [52, 53].

Beyond transcriptomic and spatial analyses, emerging genomic and multigene expression signatures are increasingly shaping precision oncology strategies. In melanoma, several validated genomic signatures, including immune‐related profiles (e.g., IFNγ, CD8A, and PD‐L1), proliferation and differentiation markers (e.g., MITF and PRAME), and invasion‐associated pathways (e.g., AXL and extracellular matrix genes), have demonstrated prognostic and predictive value for immunotherapy response [54, 55]. These signatures converge on key biological axes, including tumor differentiation, immune activation, and cellular plasticity, and may provide a structured framework for integrating AI‐driven modeling with RNA vaccine design and CAR‐T target selection [38, 56].

The integration of such genomic signatures with AI‐based or computational models offers the potential to refine patient stratification, identify optimal neoantigen targets, and predict therapeutic response with greater accuracy. However, standardization, external validation, and cross‐platform reproducibility remain critical challenges for their routine clinical implementation.

## 10. Regulatory and Ethical Considerations

As advanced therapies converge, regulatory frameworks must evolve to ensure safety, transparency, and efficacy. The approval process for AI‐driven cancer therapies presents unique challenges, including data traceability, algorithmic explainability, and patient privacy protection [45, 57]. Ethical concerns related to equitable access, informed consent, and long‐term monitoring of patients receiving AI‐supported biologics remain critical to address [46]. Collaborative efforts among industry leaders, regulatory agencies, and ethical boards will be essential to overcome these barriers and ensure patient‐centered innovation [58–60].

In recognition of these emerging complexities, regulatory authorities are adapting their oversight strategies. In 2024, China’s National Medical Products Administration (NMPA) released draft guidelines for AI‐integrated biologics, emphasizing data integrity, continuous model retraining, and algorithmic explainability. Similarly, the U.S. Food and Drug Administration (FDA) [61], through its Digital Health Center of Excellence, and the European Medicines Agency (EMA) have also issued a reflection paper on the use of AI across the medicinal product lifecycle, focusing on validation, data governance, and explainability [62]. At the global level, frameworks from the World Health Organization and the Organization for Economic Co‐operation and Development emphasize ethical AI deployment, including fairness, accountability, and responsible data governance [63, 64]. These initiatives collectively aim to harmonize ethical and technical oversight while facilitating safe and responsible innovation in oncology.

Beyond transparency and validation, ethical frameworks must include bias mitigation, data sovereignty, and global accessibility. Bias mitigation requires algorithmic audits using demographically balanced datasets and fairness metrics before clinical deployment. Data sovereignty ensures that genomic information from underrepresented populations remains governed locally, aligning with WHO and Organization for Economic Co‐operation and Development (OECD) recommendations for ethical AI in healthcare. Finally, equitable access to AI‐enabled biologics demands cost‐sharing strategies and open‐source training frameworks that democratize technology diffusion in low‐ and middle‐income regions [45, 46].

## 11. Conclusion: A Vision for Future Oncology

The convergence of AI, RNA‐based vaccines, and CAR‐T cell therapies represents a promising frontier in precision oncology. By integrating computational intelligence with advanced immunotherapeutic strategies, this approach has the potential to enhance treatment personalization, improve immune targeting, and address tumor heterogeneity.

While early clinical and translational evidence supports selected components of this integrative framework, its full implementation remains in evolution. In particular, the concept of real‐time adaptive therapeutic recalibration, although conceptually compelling, should be considered an aspirational goal rather than a near‐term clinical reality pending further validation.

Future progress will depend on rigorous clinical evaluation and methodological standardization. Key priorities for clinical translation include prospective randomized trials to validate efficacy, the development of standardized benchmarks for AI model performance, and the establishment of robust regulatory and ethical frameworks to ensure safe implementation. In addition, addressing disparities in data access and ensuring the equitable availability of advanced therapies will be essential to avoid widening global healthcare inequalities.

With continued innovation, interdisciplinary collaboration, and responsible implementation, integrative oncology strategies may progressively shift cancer care from reactive treatment toward more predictive and adaptive therapeutic paradigms.

**Nomenclature**
AI: Artificial intelligence
ARR: Absolute risk reduction
CAR‐T: Chimeric antigen receptor T cell
CD4^+^/CD8^+^: Cluster of differentiation 4/8
HR: Hazard ratio
ICI: Immune checkpoint inhibitor
LNP: Lipid nanoparticle
ORR: Objective response rate
RFS: Recurrence‐free survival.

## Author Contributions

Diala Haykal conceptualized and drafted the manuscript. Brigitte Dréno contributed to critical revision and scientific validation.

## Funding

This research received no external funding.

## Disclosure

All authors reviewed and approved the final version. “AI‐based” refers specifically to machine‐learning (ML) or deep‐learning (DL) methodologies. Other entries described as “computational” or “in silico” reflect bioinformatic or algorithmic approaches without adaptive learning components.

## Ethics Statement

The authors declare that human ethics approval was not needed for this study.

## Conflicts of Interest

The authors declare no conflicts of interest.

## Data Availability

The data that support the findings of this study are available in the References section.
